# Neu-P11 Improves Type 2 Diabetes Mellitus Immune Function by Inhibiting the Hippo Signaling Pathway

**DOI:** 10.1155/ije/3385546

**Published:** 2025-10-13

**Authors:** Shichang Cai, Si-Ke Qi, Li-Na Mao, Liu Xie, Juan He, Ying-Zhuo Li, Xiu-Ping Li

**Affiliations:** ^1^Department of Human Anatomy, School of Basic Medical Sciences, Hunan University of Medicine, Huaihua, Hunan, China; ^2^School of Nursing, Hunan University of Medicine, Huaihua, Hunan, China; ^3^Department of Pathology and Research Office of the School of Basic Medicine, Hunan University of Medicine, Huaihua, Hunan, China; ^4^Department of Physiology, Hunan University of Medicine, Huaihua, Hunan, China; ^5^College of Laboratory Medicine, Hunan University of Medicine, Huaihua, Hunan, China

**Keywords:** hippo, immune function, Neu-P11, T2DM

## Abstract

**Objective:**

Melatonin (Mel) plays a significant role in maintaining bodily homeostasis and regulating insulin resistance (IR) associated with Type 2 diabetes mellitus (T2DM). Neu-P11 is a novel Mel receptor agonist that has been reported to play a critical role in immune function in T2DM. This study aims to investigate the impact of Neu-P11 on the immune function in individuals with T2DM and its potential regulatory pathways.

**Material and Methods:**

After inducing IR in 3T3-L1 cells, the study examined the impact of piromelatine (Neu-P11) and XMU-MP-1 (a Hippo pathway inhibitor) on the levels of Hippo pathway proteins, cell viability, extracellular glucose, and GLUT4 expression. After establishing T2DM in rats by a high-fat diet and streptomycin, the effects of Neu-P11 and XMU-MP-1 on glucose metabolism and serum levels of insulin, IgA, IgG, and IgM were investigated. Primary splenocytes isolated from experimental rats were analyzed for the number of immune cells and reactive oxygen species (ROS).

**Results:**

In our study, Mel, Neu-P11, and XMU-MP-1 reduced the levels of phospho-MST1/2, phospho-LATS1/LATS1, phospho-YAP/YAP, and phospho-TAZ/TAZ in the Hippo pathway and enhanced cell viability and glucose uptake capability. This effect was more evident in the Neu-P11+XMU-MP-1 group. After treatment with Mel, Neu-P11, and XMU-MP-1, respectively, T2DM rats showed slower weight gain and a decreased spleen index, suppressed splenic ROS, downregulated phosphorylated Hippo pathway proteins, decreased IgA, and increased IgG and IgM, with improved glucose and insulin tolerance. Mel, Neu-P11, and XMU-MP-1 increased the immune cell number (CD3+, CD16+, and CD19+) in T2DM rats. Notably, co-treatment of Neu-P11 and XMU-MP-1 demonstrated superior restoration across all parameters, indicating the efficacy of combinatorial targeting.

**Conclusion:**

Neu-P11 improves immune function and increases insulin sensitivity in T2DM by inhibiting the Hippo signaling pathway, offering a novel therapeutic avenue for T2DM.

## 1. Introduction

Diabetes mellitus (DM) has emerged as an increasingly prevalent chronic metabolic disorder globally [1]. Since 1980, the number of cases has almost doubled, and the prevalence of diabetes continues to rise, with projections indicating a further increase by 2025 [2]. Type 2 DM (T2DM) is marked by reduced insulin sensitivity, leading to insulin resistance (IR) and impaired blood sugar regulation, resulting in chronic hyperglycemia [3]. Diabetes can alter bone marrow composition, cellular aging, and lymphoid hematopoiesis by promoting myeloid differentiation while suppressing lymphoid differentiation [4, 5]. It weakens the immune response of patients, posing a severe threat to their health [6]. Studying the mechanisms behind changes in the immune function of diabetic patients can, therefore, help to improve patient immunity and enhance their quality of life.

Melatonin (Mel), primarily produced and released by the pineal gland, plays a regulatory role in glucose homeostasis and the development of IR in T2DM [7]. However, after entering the human body, it has a short half-life, low bioavailability, pharmacokinetic variability, potential side effects, and difficulty in extraction and synthesis. It can produce side effects at high dosages. Piromelatine (development code Neu-P11; N-[2-(1,6,7,8-tetrahydro-2H-indeno[5,4-∗b∗]furan-8-yl)ethyl]acetamide) is a novel Mel receptor agonist that offers several advantages over Mel. It has a longer-lasting effect, higher selectivity, fewer side effects, and is easily synthesized ex vivo. Neu-P11 shows promise in improving insulin sensitivity and controlling blood glucose levels [8]. Building on previous research, we found that Neu-P11 can enhance the sensitivity of IR adipocytes and increase sugar consumption. At the same time, Neu-P11 can improve insulin resistance; augment the content of immune cells, cluster of differentiation 19-positive (CD19+), cluster of differentiation 3-positive (CD3+), and natural killer (NK) cells; and strengthen humoral immunity in the peripheral blood of T2DM rats [9, 10]. In addition, Neu-P11 has been observed to increase the serum Immunoglobulin G (IgG) and Immunoglobulin M (IgM) levels, providing long-term immunity in diabetic rats, improving inflammation, and exhibiting characteristics similar to those of Mel [11]. In light of these findings, further investigation is being conducted into the mechanism by which Neu-P11 enhances immune function in T2DM rats. This study could offer a novel therapeutic approach and a target for diabetes prevention and treatment.

The Hippo signaling pathway, a key regulator of cell proliferation and apoptosis, also modulates immune cells such as macrophages, T cells, and dendritic cells [12, 13]. Crucially, the pathway helps maintain physiological homeostasis by resisting disruptions caused by pathogenic microorganisms [14, 15]. Beyond its established link to tumorigenesis and progression, the Hippo pathway is increasingly implicated in metabolic diseases [14]. The Hippo signaling pathway functions as a kinase cascade, wherein core components, Mammalian STE20-like protein kinases 1 and 2 (MST1/2, also known as STK3/4), and Large tumor suppressor 1 and 2 (LATS1/2) remain cytoplasmic and form inhibitory complexes when phosphorylated. Phosphorylation activates the pathway, leading to the phosphorylation of downstream effectors [16]. Conversely, in the inactive/suppressed state, reduced phospho-LATS (p-LATS1/2) and phospho-MST (p-MST1/2) levels enable the transcriptional co-activator Yes-associated protein 1 (YAP) to translocate to the nucleus, driving the expression of target genes [17]. In addition, XMU-MP-1, a selective small-molecule inhibitor targeting the core Hippo pathway kinases MST1 and MST2, has been widely employed in animal model studies [18].

Existing studies confirm that Mel agonists can regulate multiple metabolic diseases by modulating the Hippo signaling pathway. In polycystic ovary syndrome (PCOS), ovarian fibrosis and stiffness are mediated by the Hippo pathway, while exogenous Mel therapy improves serum hormone levels, hyperandrogenism, IR, and ovarian histopathology [19]. Ramelteon and other Mel receptor agonists alleviate bleomycin-induced pulmonary fibrosis by blocking the nuclear translocation and expression of the core Hippo molecule YAP1 [20]. Furthermore, Mel inhibits LATS1-Mps One Binder kinase activator 1 (MOB1)-YAP phosphorylation, activates the Hippo pathway, and upregulates downstream target genes Cysteine-rich angiogenic inducer 61 (Cyr61) and connective tissue growth factor (CTGF) [21]. Previous research on the Mel receptor agonist Neu-P11 focused on its improvements to glucose metabolism, body fat distribution, and insulin sensitivity, overlooking its immunomodulatory potential. Our study first links Neu-P11 to the Hippo-immune axis, systematically exploring its role within the metabolic–immune–inflammatory network in T2DM, providing a novel perspective for broadening Neu-P11's nonmetabolic therapeutic applications.

In diabetes, MST1 deficiency enhances β-cell survival and proliferation, improves blood glucose control and insulin secretion, and preserves islet structure [22]. These results align with evidence that hyperglycemia-induced Hippo pathway overactivation triggers β-cell apoptosis and impairs insulin secretion [23]. Crucially, pharmacological targeting of the Hippo signaling pathway preserves β-cell function in T2DM by modulating the activity of its core kinase components (MST1/2 and LATS1) [24]. Notably, Mel protects against diabetic cardiomyopathy by suppressing MST1 activity and restoring mitochondrial autophagy [25, 26]. Building on this foundation, we investigate through in vitro and in vivo experiments whether Neu-P11 modulates T2DM-associated immune dysfunction via Hippo signaling regulation.

## 2. Materials and Methods

### 2.1. Drugs and Chemicals

Mel (M8600, China) and streptomycin (STZ) (S8050, China) were purchased from Beijing Solarbio Science & Technology Co., Ltd. Neu-P11 (HY-105285, China) and XMU-MP-1 (HY-100526, China) were purchased from MedChemExpress BioTech Co.

Mel was dissolved in sterile distilled water to a final concentration of 1 mg/mL. Neu-P11 was uniformly suspended in saline to a final concentration of 2 mg/mL. XMU-MP-1 was dissolved in saline to a final concentration of 0.10 mg/mL. All solutions were freshly prepared before administration.

### 2.2. Cell Culture and Handling

3T3-L1 cells (ZQ0089, Zhongqiaoxinzhou, China), a mouse embryo fibroblast cell line, were cultured in Dulbecco's modification of Eagle's medium (DMEM) (D5796, Sigma, USA) with 10% fetal bovine serum (FBS) (10099141, Gibco, USA) at 37°C with 5% CO_2_ until reaching 80%–90% confluence. The 3T3-L1 preadipocyte differentiation protocol was as follows [8, 27, 28]: Preinduction: Cells were cultured until 90%–95% confluency. Induction phase (Days 0–2): Cells were treated with 1 µM dexamethasone, 0.5 mM 3-isobutyl-1-methylxanthine, and 10 µg/mL insulin in DMEM supplemented with 10% FBS. Maintenance phase (Days 2–4): The medium was replaced with DMEM supplemented with 10% FBS containing 10 µg/mL insulin and refreshed every 48 h. Maturation phase (Days 10–14): Cells were maintained in medium until then. Validation of differentiation: The successful differentiation of cells was confirmed by > 90% lipid droplet accumulation, as observed via Oil Red O staining.

Following differentiation, 3T3-L1 adipocytes were randomly divided into the following experimental groups: IR, Mel, Neu-P11, XMU-MP-1, and XMU-MP-1 + Neu-P11. In the IR group, 1% FBS, 1% BSA, 25 mmol/L, and 1 μmol/L insulin were added to the DMEM for cell culture. In the Mel group, cells were treated with 10 nmol/L Mel for 6 h [29]. In the Neu-P11 group, cells were intervened with 10 nmol/L Neu-P11 for 6 h [29]. In the XMU-MP-1 group, cells were intervened with 5 μmol/L XMU-MP-1 for 6 h [30]. In the XMU-MP-1 + Neu-P11 group, cells were intervened by 5 μmol/L XMU-MP-1 and 10 nmol/L Neu-P11. In the control group, 3T3-L1 cells were untreated.

### 2.3. Western Blotting

Cell lysates were prepared using radioimmunoprecipitation assay (RIPA) buffer (AWB0136, Abiowell, China). After incubating on ice for 10 min, the cell lysate was centrifuged at 12,000 rpm for 5 min at 4°C in a cold centrifuge (H1650R, Hunan Xiang Instrument, China), and new tubes were transferred to the supernatant. The BCA test determined protein content. The supernatant was diluted to 10 μg/μL with RIPA buffer and then mixed with 4× loading buffer at a 1:3 ratio. The mixture was boiled for 5 min, cooled on ice, and then centrifuged at 12,000 rpm for 5 min. The resulting supernatant was transferred to a new tube, divided into aliquots, and stored at −80°C. 60 μg of protein per well was loaded, followed by half-dry transfer to an nitrocellulose (NC) membrane after SDS-PAGE gel electrophoresis. 5% defatted milk powder was used for sealing at room temperature for 90 min. We diluted the primary antibody according to Table 1with 1× PBST (AWI0130, Abiowell, China). The membrane and primary antibody were co-incubated overnight at 4°C. After incubating with the secondary antibody at room temperature on a shaker for 2 h, the membranes were rinsed with 1 × TBST for 15 min three times. Subsequently, the membrane was exposed and photographed in a chemiluminescent imaging system (ChemiScope6100, Clinx Science Instruments, China), followed by analysis using ImageJ.

### 2.4. Animals

Male Sprague–Dawley (SD) rats (6–8 weeks old, 180–220 g) were sourced from Hunan Slake Jingda Animal Experimental Company. The rats were housed in a specific pathogen-free (SPF) environment with controlled conditions (temperature: 20°C–24°C, humidity: 40%–50%), a 12 h light/12 h dark cycle, and provided with standard rodent chow and water ad libitum. All experimental procedures and animal handling were performed with the approval of the Animal Care and Use Committee of the Hunan University of Medicine, Hunan University of Medicine (No. 2024-A09002), and in accordance with the National Institutes of Health Guide for the Care and Use of Laboratory Animals, and studies involving laboratory animals follow the ARRIVE guidelines.

### 2.5. Experimental Protocols

After 1 week of acclimatization on standard chow, 18 rats were randomly divided into a control group (*n* = 3) receiving standard diet and a model group (*n* = 15) fed a high-fat diet (HFD). The HFD was obtained from Jiangsu Xietong Bio-Engineering Co., Ltd (China). After 4 weeks of dietary intervention, the rats in the model group were intraperitoneally injected with a 1% STZ solution at a dose of 30 mg/kg (interval, 2 days), while the control group received an intraperitoneal injection of citric acid buffer at the same dose [29]. One week postinjection, random tail vein blood glucose ≥ 250 mg/dL (13.9 mmol/L) and fasting blood glucose (FBG) ≥ 11.1 mmol/L were determined as a standard for T2DM rats.

The T2DM rats (*n* = 15) were randomly allocated into five subgroups (3 animals/group): In the model group, rats were treated with normal saline. In the Mel group, rats were gavaged with 10 mg/kg Mel; in the Neu-P11 group, rats were gavaged with 20 mg/kg Neu-P11 [11]; in the XMU-MP-1 group, rats were intraperitoneally injected with 1 mg/kg XMU-MP-1; in the XMU-MP-1 + Neu-P11 group, rats were intraperitoneally injected with 1 mg/kg XMU-MP-1 and gavaged with 20 mg/kg Neu-P11 [18]. Each group was intervened once a day at regular intervals for 21 days; in the control group, rats received a normal diet and equivalent volumes of distilled water or saline via gavage.

The experimental setup process is shown in Figure S1.

### 2.6. Cell Counting Kit-8 (CCK8) Assay

Cells were seeded in 96-well plates at a density of 5 × 10^3^ cells per well in 100 μL. Three replicate wells were incubated, followed by treatment as described. Subsequently, each well was exposed to a 10% CCK8 working solution (HY-K0301, eBioscience, USA) at 37°C and 5% CO_2_ for 4 h, and the optical density (OD) was measured at 450 nm using a microplate reader (MB-530, Huisong, China).

### 2.7. Reverse Transcription–Quantitative Polymerase Chain Reaction (RT-qPCR)

Total RNA was isolated from cultured cells using the Trizol total RNA extractor method (15596026, Thermo, USA). The density and purity of RNA were estimated spectrophotometrically. The cDNA was synthesized using a reverse transcription kit (CW2569, CWBIO, China). RT-qPCR reactions were performed on a QuantStudio 1 RT-PCR instrument from Applied Biosystems, using the Ultra SYBR Mixture (CW2601, CWBIO, China) according to the manufacturer's instructions. The expression levels of the genes were determined using the 2^−ΔΔCt^. The gene-specific primers were designed using Primer Premier 5.0 software, with their sequences and specifications detailed in Table 2.

### 2.8. Isolation of Primary Splenocytes

The spleen tissues were obtained from rats and processed using sterile tools, including cutting and grinding in a 100 mm Petri dish with RPMI-1640 medium. The tissue fragments were washed with a culture medium, passed through a sieve, and treated with an erythrocyte lysis solution. We filter the suspension through a 70 μm cell strainer to remove large tissue debris. Then, the filter was centrifuged at 1000 rpm for 5 min to obtain suspensions of mononuclear cells from splenic tissue. Subsequently, cells were resuspended and identified using flow cytometry. Lastly, splenocytes were incubated in DMEM dishes containing 10% FBS as well as 1% STZ-penicillin at 37°C in 5% CO_2_.

### 2.9. Detection of Reactive Oxygen Species (ROS)

2′,7′-Dichlorodihydrofluorescein diacetate (DCFH-DA) was diluted 1:1000 with serum-free culture medium to achieve a final concentration of 10 µM. The digested tissue residue was then treated with the diluted DCFH-DA solution. Subsequently, the digested tissue solution was filtered through a cell strainer to obtain a cell suspension. The cells were then allowed to incubate at 37°C for 20 min. Following this, the cells were washed three times with serum-free cell culture medium, trypsin-digested, and collected for analysis using flow cytometry (A00-1-1102, Beckman, USA).

### 2.10. Flow Cytometry

The digested cells were washed twice with PBS. The cells were resuspended in 100 μL basal medium and stained with antibodies against CD3+ (25-0038-42, eBioscience, USA), CD16+ (25-0161-82, eBioscience, USA), and CD19+ (12-0199-42, eBioscience, USA) for a wrap in aluminum foil. After the 30-min incubation period, the cells were washed twice with PBS, resuspended in 200 µL of staining buffer, and analyzed using flow cytometry (A00-1-1102, Beckman, USA).

### 2.11. Body Weight, FBG, and Spleen Index Measurement

FBG and body weight of rats were measured at 6 h after feeding at the same time on Days 0, 7, 14, and 21, respectively. The rats were humanely euthanized by injecting 150 mg/kg pentobarbital intraperitoneally. The spleens were extracted from rats, then weighed, and recorded. The spleen index was computed as the spleen weight (mg) to body weight (g) ratio.

### 2.12. Enzyme-Linked Immunosorbent Assay (ELISA)

Blood samples were incubated at room temperature for 2 h and then centrifuged at 1000 g for 15 min at 2°C–8°C. The resulting supernatant was transferred to a new 1.5 mL centrifuge tube for subsequent analysis. Three standard wells, 3 wells for samples to be tested, and 3 blank wells (without adding any solution) should be set up in a 96-well plate. Each well should be filled with 50 μL of standard or samples to be tested. Then, each well was followed by the addition of 50 μL of the enzyme conjugate (without the addition to the blank wells), IgA, IgM (CSB-E0798r, CUSABIO, China), and IgG (CSB-E07981r, CUSABIO, China). The contents of the wells were mixed gently by shaking. After that, the plate should be covered with a sealing film and incubated at 37°C for 60 min. The liquid in the wells should be discarded. The plate should be washed five times. Each wash should last for 2 min, with 200 μL of solution added to each well, before the plate is shaken to remove any remaining liquid.

We added 90 μL of the substrate solution to each well and incubated the plate at 37°C for 20 min in the dark. Then, 50 μL of the termination solution was added to each well to stop the reaction. We measured the OD value of each well at 450 nm using an enzyme marker.

### 2.13. Glucose Tolerance Tests (GTTs) and Insulin Tolerance Tests (ITTs)

On Day 14 of the experiment, all rats, after a 6 h fast, were subjected to a GTT by administering a 50% glucose solution (2.5 g/kg). Blood specimens were collected to evaluate blood glucose levels at 0, 30, 60, and 120 min after the administration of the drug. On Day 21, an ITT was performed. The rats were fasted for 6 h before receiving an insulin injection at a dose of 0.5 U/kg. Blood samples were collected from the rats at 0, 30, 60, and 120 min postinjection for blood glucose level measurements.

### 2.14. Statistical Analyses

Statistical analysis was performed using GraphPad Prism 8.0. Data with a normal distribution are presented as mean ± standard deviation. Student's *t*-test was utilized for comparisons between two groups. Data were analyzed using one-way ANOVA followed by Tukey's post hoc test for single-factor comparisons, or two-way ANOVA followed by Šidák's multiple comparisons test for multifactor analyses. Statistical significance was set at *p* < 0.05. All experiments were conducted thrice, yielding consistent results.

## 3. Results

### 3.1. Neu-P11 Regulates the Hippo Signaling Pathway in IR Adipocytes

To investigate the effect of Neu-P11 in IR adipocytes, we induced differentiation into adipocytes by culturing mouse 3T3-L1 cells [31]. Oil Red O staining solution was used to detect the staining of the cells after induced differentiation. The image analysis system was used to capture the image.  Figure S2A shows the normal morphology of 3T3-L1 cells before staining. After staining, the intracellular fat of 3T3-L1 cells turned red. The results are shown in Figure S2B.

**Figure 1:**
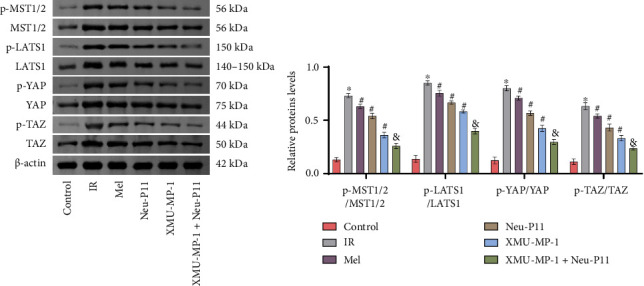
Neu-P11 regulates the Hippo signaling pathway in insulin-resistant adipocytes. The relative protein levels of p-MST1/2/MST1/2, p-LATS1/LATS1, p-YAP/YAP, and p-TAZ/TAZ in 3T3-L1 cells were determined by western blotting. ^∗^*p* < 0.05 compared to the control group, ^#^*p* < 0.05 relative to the IR group, and ^&^*p* < 0.05 against the Neu-P11/XMU-MP-1 group.

The Hippo signaling pathway has a significant influence on the differentiation of 3T3-L1 cells into adipocytes [32]. P-MST1/2, MST1/2, p-LATS1, LATS1, p-YAP, YAP, p-TAZ, and TAZ are protein molecules associated with the Hippo signaling pathway [33, 34]. To investigate the implications of Neu-P11 on the Hippo signaling pathway in IR adipocytes, we obtained the Hippo signaling pathway inhibitor XMU-MP-1. Western blotting experiments were performed to determine the levels of p-MST1/2/MST1/2, p-LATS1/LATS1, p-YAP/YAP, and p-TAZ/TAZ in the IR group, all significantly increased compared to the control group (Figure 1). These results confirmed that the Hippo signaling pathway was highly expressed in IR adipocytes. The expression of proteins in the Hippo signaling pathway was lower in the Mel group, the Neu-P11 group, and the XMU-MP-1 group compared to the IR group. The protein expression of the XMU-MP-1 + Neu-P11 group was lowest compared to the XMU-MP-1 group, the Neu-P11 group, and the Mel group. These results suggest that Neu-P11 may regulate the Hippo signaling pathway in IR adipocytes.

### 3.2. Neu-P11 Regulates Glucose Uptake in IR Adipocytes via the Hippo Signaling Pathway

**Figure 2:**
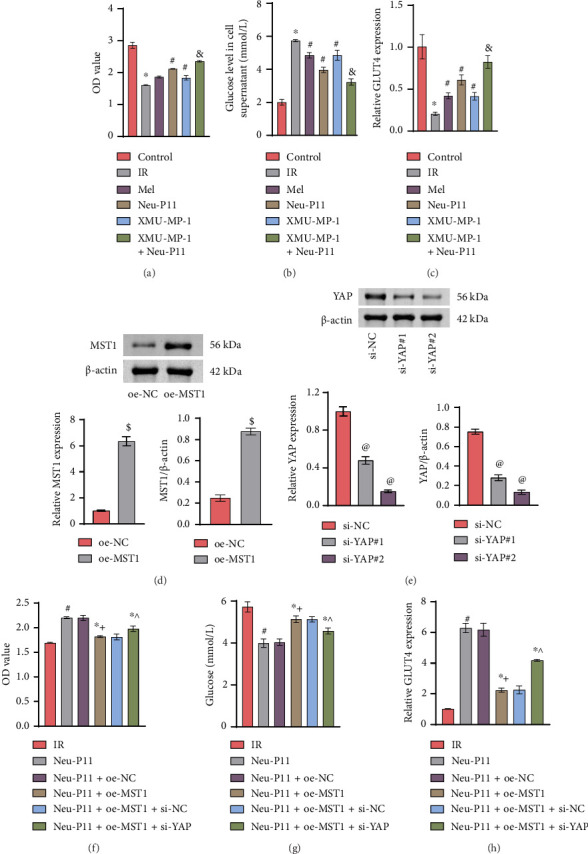
Neu-P11 regulates glucose uptake in adipocytes through the Hippo signaling pathway. (a) The cell viability of six groups of cells was evaluated with the CCK8 assay. (b) The glucose assay kit employed the oxidase method to measure the glucose levels in the cell supernatant. (c) The relative expression of GLUT4 was detected by RT-qPCR. (d) MST1 expression was analyzed by RT-qPCR and western blotting. (e) YAP expression was assessed by RT-qPCR and western blot. (f) Cell viability was assessed using the CCK8 assay. (g) Extracellular glucose levels were measured in cell culture supernatants using a glucose oxidase assay kit. (h) Relative GLUT4 expression was quantified by RT-qPCR. ^∗^*p* < 0.05 vs. the control group, ^#^*p* < 0.05 vs. the IR group, and ^&^*p* < 0.05 vs. the Neu-P11/XMU-MP-1 group, ^$^*p* < 0.05 vs. the oe-NC group, ^@^*p* < 0.05 vs. the si-NC group, ^∗+^*p* < 0.05 vs. the Neu-P11 + oe-NC group, and ^∗^^*p* < 0.05 vs. the Neu-P11 + oe-MST1 + si-NC group.

To explore Neu-P11's impact on IR adipocytes via the Hippo signaling pathway, cell viability was assessed using a CCK8 assay, and glucose levels in the cell supernatant were measured using ELISA. The expression of the Glucose transporter 4 (GLUT4) was determined by RT-qPCR. Results demonstrated relative to the control group, cell viability was reduced in the IR group, and cellular glucose uptake capacity and the expression of GLUT4 were lower. When it was compared to the IR group, cell viability was enhanced in the Mel group, Neu-P11 group, XMU-MP-1 group, and the XMU-MP-1+Neu-P11 group, and cellular glucose uptake capacity and expression of the GLUT4 were increased. Compared to the Neu-P11 group and the XMU-MP-1 group, cell viability and cellular glucose uptake ability were greater, and the expression of the GLUT4 was higher in the XMU-MP-1 + Neu-P11 group (Figures 2(a), 2(b), and 2(c)). Next, we established stable cell lines overexpressing MST1 or with YAP knockdown (Figures 2(d) and 2(e)). Subsequent analysis revealed that, compared to the IR group, the Neu-P11-treated group exhibited increased cell viability and GLUT4 expression, along with decreased glucose levels in the supernatant, indicating that Neu-P11 enhanced cellular glucose uptake. Furthermore, cell viability and GLUT4 expression were inhibited, while supernatant glucose levels increased in the Neu-P11+oe-MST1 group relative to the Neu-P11+oe-NC group. Importantly, YAP knockdown attenuated the effects of MST1 overexpression (Figures 2(f), 2(g), and 2(h)). Collectively, these data demonstrate that Neu-P11 regulates glucose uptake in IR adipocytes via the Hippo signaling pathway. Those results confirm that Neu-P11 resists adipocyte glucose uptake by regulating the Hippo signaling pathway IR.

### 3.3. Neu-P11 Modulates the Hippo Signaling Pathway in Rats With T2DM

**Figure 3:**
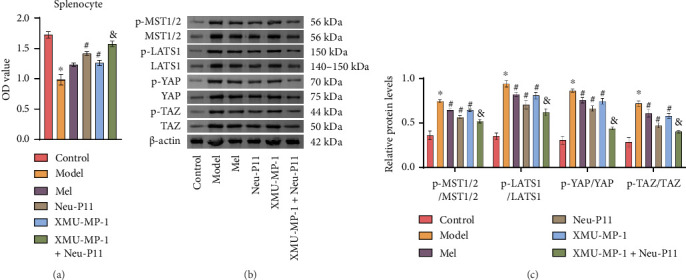
Neu-P11 modulates the Hippo signaling pathway in rats with T2DM. (a) Viability of splenic cells was evaluated with the CCK8 assay. (b, c) Western blotting assay assessed the levels of p-MST1/2/MST1/2, p-LATS1/LATS1, p-YAP/YAP and p-TAZ/TAZ in splenocytes. ^∗^*p* < 0.05 compared to the control group, ^#^*p* < 0.05 relative to the model group, and ^&^*p* < 0.05 against the Neu-P11/XMU-MP-1 group.

Following the animal experiment, splenocytes were isolated from the spleen tissue of each group of rats. The CCK8 assay was performed to assess the cell viability of the splenocytes. Western blotting analysis revealed altered expression levels of p-MST1/2, MST1/2, p-LATS1/LATS1, p-YAP/YAP, and p-TAZ/TAZ in different experimental groups. The model group exhibited decreased cell viability and elevated levels of these molecules compared to the control group. Conversely, the Mel, Neu-P11, and XMU-MP-1 groups showed increased cell viability and reduced levels of these molecules relative to the model group. Furthermore, when relative to the XMU-MP-1 group and Neu-P11 group, the XMU-MP-1+Neu-P11 group demonstrated even higher cell viability and lower levels of p-MST1/2/MST1/2, p-LATS1/LATS1, p-YAP/YAP, and p-TAZ/TAZ (Figures 3(a), 3(b), and 3(c)). These findings indicate that Neu-P11 may regulate the Hippo signaling pathway. The observed effects align with the outcomes of previous cell experiments, supporting the notion that Neu-P11 can effectively regulate the Hippo signaling pathway in T2DM rats.

### 3.4. Neu-P11 Improves IR and Glucose Homeostasis via the Hippo Signaling Pathway in T2DM Rats

**Figure 4:**
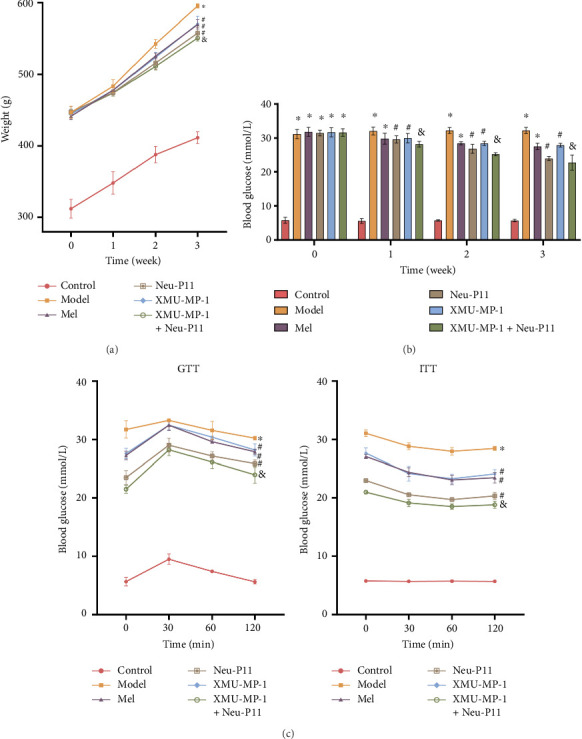
Neu-P11 improves insulin resistance and glucose balance in T2DM rats through the Hippo signaling pathway. (a) Weight curve of rats in six groups at 21 days. (b) Fasting blood glucose trend in rats of six groups at 21 days. (c) Measurement of blood glucose and insulin levels in rats during the 0, 30, 60, and 120 min intervals after GTT and ITT after oral glucose administration (2 g/kg). ^∗^*p* < 0.05 compared to the control group, ^#^*p* < 0.05 relative to the model group, and ^&^*p* < 0.05 against the Neu-P11/XMU-MP-1 group.

To elucidate the relationship between Neu-P11 and the Hippo signaling pathway in T2DM rats, we monitored the body weight of the rats and quantified their FBG and insulin profiles throughout the experiment on Days 7, 14, and 21 (with Day 0 designated as the first day of administration). Experiment results revealed that the rats in the model group experienced a substantially quicker weight gain and increased FBG concentration compared to the control group. Conversely, rats in the Mel group, XMU-MP-1 group, and Neu-P11 groups exhibited a slower rate of weight gain and a reduction in FBG levels compared with the model group. When we compared the XMU-MP-1 group and Neu-P11 group to the XMU-MP-1 + Neu-P11 group, the rats in the latter demonstrated slower weight gain and lower FBG levels (Figures 4(a) and 4(b)). After oral glucose administration (2 g/kg), we measured the blood glucose and insulin levels of the T2DM rats at 0, 30, 60, and 120 min. The findings revealed that rats in the model group displayed significantly increased glucose and insulin levels compared to those in the control group. However, the Mel group, XMU-MP-1 group, and Neu-P11 group had noticeably lower glucose and insulin levels compared to the model group. Notably, the XMU-MP-1 + Neu-P11 group demonstrated an even further reduction in glucose and insulin levels (Figures 4(c)). These results confirm that Neu-P11 enhances insulin sensitivity and glucose balance in T2DM rats. Those experiments highlight that the Hippo signaling pathway is significant in these mechanisms.

### 3.5. Neu-P11 Enhances Immune Responses in T2DM Rats Through a Hippo Signaling Pathway

**Figure 5:**
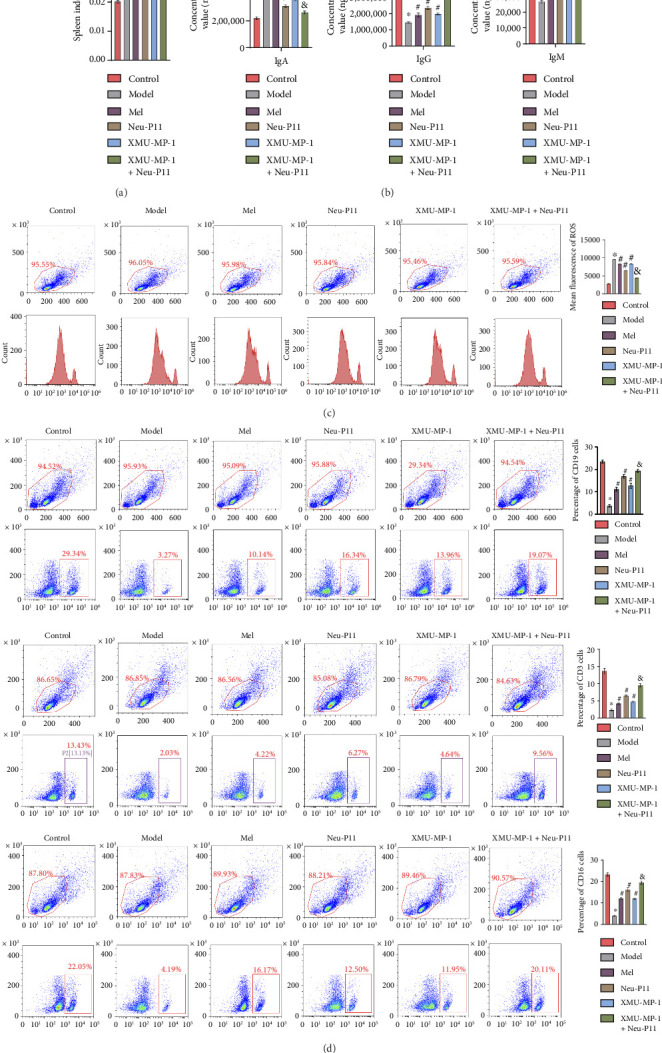
Neu-P11 improves immune function in T2DM rats through the Hippo signaling pathway. (a) The spleen index of rats in each group. (b) ELISA was utilized to measure serum levels of IgA, IgG, and IgM in all groups. (c) ROS was detected by flow cytometry in splenic tissue. (d) Flow cytometry was employed to assess changes in the number of immune T cells (CD3+), NK cells (CD16+), and B cells (CD19+) in all groups. Statistical significance was denoted as ^∗^*p* < 0.05 compared to the control group, ^#^*p* < 0.05 relative to the model group, and ^&^*p* < 0.05 against the Neu-P11/XMU-MP-1 group.

Existing studies have established that ROS can trigger oxidative lesions of intracellular lipids, proteins, and nucleic acids. This leads to lipid peroxidation in cell membranes, protein degradation, and DNA damage, instigating apoptosis or mutation and hastening cellular aging and disease onset [34, 35]. In this study, we used flow cytometry to measure ROS levels, immune cell counts, and evaluate the spleen index in rats.  Figure 5(a) indicated a decreased spleen index in T2DM rats treated with Mel, XMU-MP-1, or Neu-P11 alone, and the combination of XMU-MP-1 and Neu-P11 induced a more pronounced reduction versus monotherapies. Flow cytometry results showed that compared to the model group, the level of ROS was decreased and accompanied by an increase in the number of CD3+, CD16+, and CD19+ cells in the Mel, XMU-MP-1, and Neu-P11 groups. Notably, the dual-treatment of XMU-MP-1 and Neu-P11 showed further ROS reduction and higher immune cell counts than single-agent (Figures 5(c) and 5(d)). Those results highlight that Neu-P11 has potential as an immune function enhancer in T2DM rats via a Hippo signaling pathway.

We used the ELISA to evaluate the concentrations of IgA, IgM, and IgG in rat serum from each group. The results showed a surge in IgA levels and a decrease in IgM and IgG levels in the model group over the control group (Figure 5(b)). However, in the Mel group, Neu-P11 group, and XMU-MP-1 group, serum IgA levels diminished, whereas IgM and IgG levels increased as compared to the model group. Moreover, the serum levels of IgA were decreased, while IgG and IgM were increased in the XMU-MP-1 + Neu-P11 rat group in comparison to the XMU-MP-1 group and the Neu-P11 group. In summary, these findings collectively suggest that Neu-P11 can mitigate ROS production and enhance immune function. At the same time, these experiments reveal that Neu-P11 can alleviate the inflammatory response in T2DM rats by manipulating the Hippo signaling pathway.

## 4. Discussion

T2DM represents around 90% of the worldwide diabetic population, with its prevalence escalating continuously [36]. The primary pathological hallmarks of T2DM include deteriorated β-cell function and diminished insulin sensitivity. These can result in immune system dysregulation and tissue damage, subsequently inducing IR, organ failure, premature aging, and intensified organ damage [37, 38]. Thus, enhancing immune functions in T2DM patients is of paramount importance.

Traditionally, Mel has been primarily associated with the regulation of sleep cycles and biological rhythms. However, recent studies have revealed its broader roles in modulating immune cell differentiation and function, as well as its antioxidant and anti-inflammatory properties [39–41]. Neu-P11, a novel Mel receptor agonist, demonstrates advantages over Mel, including prolonged duration of action, high selectivity, fewer adverse effects, and facile in vitro synthesis. Previous research has indicated that Neu-P11 normalizes lipid and glucose metabolism, suggesting therapeutic potential for T2DM [8]. In T2DM rats, Neu-P11 treatment alleviated symptoms of hyperphagia, polydipsia, hyperglycemia, impaired glucose tolerance, and IR compared to untreated models, and the levels of glucose following ingestion in rats were reduced by 12.98%, 12.26%, and 14.58%, respectively, at 30, 60, and 120 min [9]. Li et al. reported increased proportions of T cells, NK cells, and B cells in peripheral blood; elevated IFN-γ levels; and decreased ROS and TNF-α in Neu-P11-treated rats. Their study further showed that combined Mel and Neu-P11 therapy upregulated Phosphorylated insulin receptor substrate 1 (p-IRS-1); Phosphatidylinositol 3-kinase (PI3K), which activates AKT to regulate cell survival, growth, and metabolism; and Phosphorylated glycogen synthase kinase-3 beta (p-GSK3β), which is a key downstream target of AKT, thereby relieving inhibition of multiple prosurvival and antiapoptotic pathways [29]. Thus, Neu-P11 ameliorates IR by enhancing insulin signaling pathway activity, consistent with our findings.

The Hippo signaling pathway is crucial in regulating cell development, proliferation, and immune function. It has also been found to be altered in T2DM, which may have a significant impact on cellular immune responses. Our study demonstrates that the high expression of Hippo signaling pathway–associated protein molecules in IR adipocytes promotes IR and reduces cellular glucose uptake, whereas Neu-P11 can mitigate this effect. Those results suggest that Neu-P11 can modulate the Hippo signaling pathway in IR cells to improve insulin sensitivity and maintain glucose homeostasis. In this study, the percentage of immune cells (CD3+, CD16+, and CD19+) was significantly higher in Neu-P11 + XMU-MP-1-treated T2DM rats. At the same time, the level of ROS was decreased. The inflammation was improved, and the spleen index of the rats was decreased even more significantly. The number of immune cells was increased even more significantly. Inflammation was improved even more significantly. These data demonstrated that Neu-P11 could regulate the Hippo signaling pathway to enhance immune function in T2DM.

Consistent with Zeng et al., who demonstrated Hippo pathway–mediated regulation of IgA, IgG, IgM, and sIgA secretion in immunosuppressed mice [42], our data revealed that Neu-P11 suppresses Hippo signaling, leading to IgA downregulation and IgG/IgM upregulation, and establishes a mechanistic axis connecting Mel agonism, Hippo modulation, and humoral immunity. While existing studies focus on the metabolic effects of Mel agonists [43], we provide the evidence that Neu-P11 confers dual immunomodulatory and metabolic benefits in T2DM, establishing its novel role in immunometabolic regulation. We speculate that the mechanism involves the activity of key molecules in the Hippo signaling pathway, such as MST, LATS, YAP, and TAZ. Those molecules can directly influence the response of immune cells, which in turn affects the pathological course of T2DM. Activated molecules, such as MST, LATS, YAP, and TAZ, can direct immune cells to produce excessive inflammatory factors, promote islet cell damage, and decrease insulin secretion, thereby exacerbating T2DM. Conversely, by inhibiting the Hippo signaling pathway, it may be possible to enhance immune function in T2DM. The above may be a potential mechanism by which Hippo regulates glucose uptake in IR adipocytes. It may also regulate IR and immune function in T2DM rats.

This study has limitations. First, human data on Neu-P11 effects are lacking. Second, efficacy comparisons with other Mel receptor agonists (e.g., ramelteon and agomelatine) in T2DM models are absent. Future research should explore nanocarrier technology to overcome the limitations of first-pass metabolism and conduct clinical trials evaluating the immunometabolic effects of Neu-P11 versus established agonists.

In summary, the findings of this study require further validation and in-depth analysis. However, our findings provide strong evidence to support the idea that Neu-P11 improves immune function in T2DM by modulating the Hippo signaling pathway and offer new ideas for the treatment and management of T2DM.

## 5. Conclusion

Our study provides evidence that Neu-P11 can regulate the Hippo signaling pathway to enhance immune function in T2DM. These findings shed new light on the treatment and care of patients with T2DM. It may offer a theoretical underpinning for subsequent in-depth investigations.

## Figures and Tables

**Table 1:** Information on the antibody.

Name	Dilution rate	Cat. number	Company	Country
MST1/2	1:500	PA5-36100	ThermoFisher	USA
p-MST1/2	1:1000	ab76323	Abcam	UK
LATS1	1:1000	ab243656	Abcam	UK
p-LATS1	1:5000	28998-1-AP	Proteintech	USA
YAP	1:2000	13584-1-AP	Proteintech	USA
p-YAP	1:2000	29018-1-AP	Proteintech	USA
TAZ	1:1000	23306-1-AP	Proteintech	USA
p-TAZ	1:1000	AF4315	Affinity	USA
β-Actin	1:5000	AWA80001	Abiowell	China

**Table 2:** The primer sequencer.

Primer	Forward 5′-3′	Reverse 5′-3′
M-GLUT4	AGCCAGCCTACGCCACCATAG	CAGCAGAGCCACGGTCATCAAG
M-β-actin	ACATCCGTAAAGACCTCTATGCC	TACTCCTGCTTGCTGATCCAC

## Data Availability

The data that support the findings of this study are available from the corresponding author upon reasonable request.

## Supporting Information

Supporting Information 1 Figure S1. Animal experiment process.

Supporting Information 2 Figure S2. Cell morphology was observed, and Oil Red O staining was performed. A. Normal cell morphology before staining under the light microscope B. The cell status after Oil Red O staining.
