# Supplementary material for: Neu-P11 Improves Type 2 Diabetes Mellitus Immune Function by Inhibiting the Hippo Signaling Pathway
Source: Int J Endocrinol. 2025 Oct 13;2025:3385546. doi: 10.1155/ije/3385546 (PMC12537237; doi:10.1155/ije/3385546)
Supplement: Supporting Information 1 — Figure S1. Animal experiment process. [file 3385546.f1.pdf]

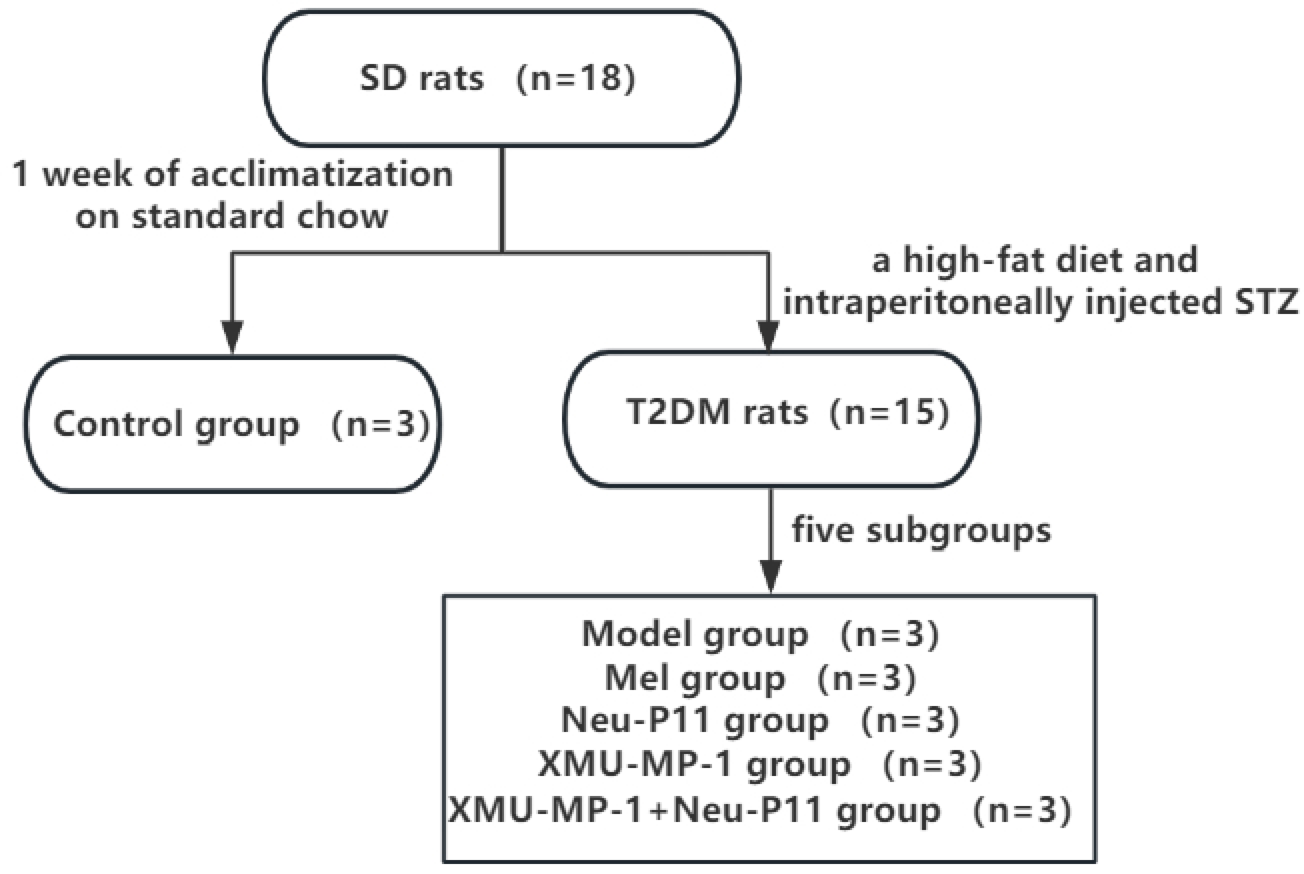
SD rats (n=18)
1 week of acclimatization on standard chow
a high-fat diet and intraperitoneally injected STZ
Control group (n=3)
T2DM rats (n=15)
five subgroups
Model group (n=3)
Mel group (n=3)
Neu-P11 group (n=3)
XMU-MP-1 group (n=3)
XMU-MP-1+Neu-P11 group (n=3)
