# Supplementary figures and images for: Neu-P11 Improves Type 2 Diabetes Mellitus Immune Function by Inhibiting the Hippo Signaling Pathway
Source: Int J Endocrinol. 2025 Oct 13;2025:3385546. doi: 10.1155/ije/3385546 (PMC12537237; doi:10.1155/ije/3385546)

**A**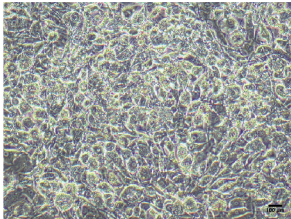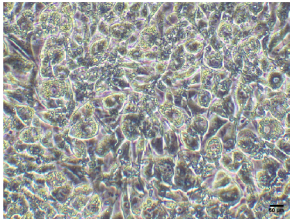**B**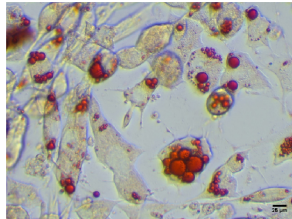

Supplement: Supporting Information 2 — Figure S2. Cell morphology was observed, and Oil Red O staining was performed. A. Normal cell morphology before staining under the light microscope B. The cell status after Oil Red O staining. [file 3385546.f2.pdf]
